# Latent Profile Analysis of Professional Quality of Life and Its Relationship With Posttraumatic Growth Among Nurses: A Cross‐Sectional Study

**DOI:** 10.1155/jonm/6550718

**Published:** 2026-07-15

**Authors:** Li Zeng, Yuan Zhang, Zhongqing Yuan, Fengxue Yang, Xiuying Hu

**Affiliations:** ^1^ Innovation Center of Nursing Research and Nursing Key Laboratory of Sichuan Province, West China Hospital, Sichuan University/West China School of Nursing, Sichuan University, Chengdu, Sichuan, China, scu.edu.cn; ^2^ Office of Academic Affairs, Neijiang Health Vocational College, Neijiang, Sichuan, China, scu.edu.cn; ^3^ School of Smart Nursing, Sichuan Nursing Vocational College, Deyang, Sichuan, China, scu.edu.cn

**Keywords:** cross-sectional study, latent profile analysis, nurses, posttraumatic growth, professional quality of life

## Abstract

**Background:**

Professional quality of life comprehensively reflects both positive and negative experiences of nurses in helping professions, yet its latent profiles and relationship with posttraumatic growth remain unclear.

**Objectives:**

To identify latent profiles of nurses’ professional quality of life and to explore the differences in posttraumatic growth across different profiles.

**Design:**

A cross‐sectional study.

**Methods:**

Nurses (*n* = 1010) from 10 tertiary general hospitals in Sichuan Province, China, were recruited from April to June 2025. Participants were asked to complete questionnaires assessing sociodemographic characteristics, professional quality of life, and posttraumatic growth. Latent profile analysis was used to identify professional quality of life profiles. Data were analyzed using univariate analysis, multivariate logistic regression, and hierarchical linear regression.

**Results:**

Three professional quality of life profiles among nurses were identified: “Depleted” (12.48%), “Balanced” (63.96%), and “Thriving” (23.56%). Profiles were associated with marital status, weekly exercise, daily sleep hours, weekly work hours, main shift, and workplace violence. Hierarchical regression showed that different profiles were significantly associated with posttraumatic growth among nurses.

**Conclusion:**

Nurses’ professional quality of life demonstrates group heterogeneity, which is associated with individual and work‐related characteristics, and professional quality of life profiles are significantly associated with posttraumatic growth.

**Implications for Nursing Management:**

The findings may enable administrators to recognize the needs of nurses in different profiles and consider targeted strategies, such as improving the work environment and providing psychological support, to foster nurses’ psychological resources and positive growth.

## 1. Introduction

The nursing profession is widely recognized as one of the most stressful occupations due to its high‐intensity demands, significant emotional involvement, and frequent exposure to traumatic events such as patient suffering and death [1, 2]. Nurses working persistently in such high‐pressure environments are not only exposed to routine occupational stressors, including excessive workloads and role conflicts, but also frequently experience direct or indirect traumatic incidents such as patient safety events and workplace violence [3, 4]. Sustained occupational stress and trauma exposure pose severe challenges to nurses’ psychological well‐being, potentially leading to a series of negative psychological outcomes such as burnout and compassion fatigue, which in turn threaten the stability of the nursing workforce and the quality of care [5, 6]. Consequently, a deeper investigation into the psychological responses and adaptation patterns of nurses when coping with occupational stress and trauma is of paramount importance for safeguarding their mental health and ensuring the sustainable development of human resources within healthcare systems.

## 2. Background

### 2.1. Posttraumatic Growth

Traditionally, research attention has predominantly been directed toward the adverse psychological consequences of occupational stress and trauma exposure among nurses, such as posttraumatic stress disorder [7]. With the rise of positive psychology, however, it has been recognized that individuals may not only experience adverse consequences but also undergo positive psychological transformation after struggling with significant adversity, referred to as posttraumatic growth [8, 9]. Posttraumatic growth theory posits that, following traumatic events, individuals may achieve growth beyond their pretrauma level through cognitive processing and meaning reconstruction [10]. For nurses, the ongoing challenges and potentially traumatic experiences encountered in daily practice (such as patient death and suffering, workplace violence, high workloads, and sustained emotional demands) can serve not only as sources of stress but also as catalysts for reflecting on life meaning, reshaping professional values, and enhancing personal resilience. Unlike a single discrete traumatic event, nurses often face repeated, cumulative adversities over time [11]. Investigating posttraumatic growth among nurses contributes to a more comprehensive understanding of their psychological adaptation outcomes and offers potential targets for interventions aimed at fostering psychological resilience and professional development.

### 2.2. Professional Quality of Life (ProQOL)

ProQOL is a multidimensional construct that comprehensively reflects both the positive and negative feelings an individual experiences in a helping profession [12]. ProQOL encompasses three core dimensions: compassion satisfaction, burnout, and secondary traumatic stress [13]. Compassion satisfaction refers to the pleasure and sense of accomplishment derived from helping others, serving as a positive, protective resource [14]. In contrast, burnout and secondary traumatic stress represent the emotional exhaustion and traumatic reactions resulting from occupational stress and trauma exposure, constituting negative risk factors [15]. According to the conservation of resources theory, individuals strive to obtain, retain, and protect their valuable resources, while stress occurs when these resources are lost or threatened with loss [16]. In this framework, ProQOL among nurses reflects patterns of personal psychological resource gain or loss within high‐pressure work environments. High compassion satisfaction is associated with resource gain, facilitating coping with stress and potentially fostering growth, whereas high burnout and secondary traumatic stress are associated with sustained resource depletion, which may hinder positive adaptation processes [15, 17]. Thus, ProQOL is a key variable associated with the psychological adaptation outcomes of nurses in high‐stress work environments, such as posttraumatic growth.

Although the relationship between nurses’ ProQOL and posttraumatic growth has been investigated [15, 17], these studies were predominantly conducted using a variable‐centered approach. Nurses were often treated as a homogeneous group when exploring relationships between variables, thereby overlooking individual heterogeneity. In reality, different nurses may exhibit distinct ProQOL profiles [12]. As a person‐centered method, latent profile analysis can identify subgroups with similar response patterns based on multiple continuous observed indicators, thereby offering deeper insights into within‐group heterogeneity [18, 19]. Unlike variable‐centered approaches that assess each ProQOL dimension separately, LPA integrates the three dimensions to capture naturally occurring configurations of resource gain and depletion within individuals. Furthermore, latent profile analysis provides a more scientifically robust approach for examining the relationship between different ProQOL profiles and other variables [12].

To our knowledge, limited research has extended a ProQOL profile framework to posttraumatic growth as an outcome among nurses. Based on the above, this study aims to apply latent profile analysis to explore the heterogeneity in nurses’ ProQOL and to examine differences in posttraumatic growth levels across identified profiles. The specific objectives are as follows: 1) to identify latent profiles of nurses’ ProQOL; 2) to compare the distribution of nurses with different characteristics across ProQOL latent profiles; 3) to describe the relationship between different ProQOL latent profiles and levels of posttraumatic growth. The findings are expected to provide a stratified reference for nursing administrators to recognize nurses with different support needs, thereby facilitating more resource‐appropriate psychological support strategies.

## 3. Methods

### 3.1. Design and Data Collection

A cross‐sectional study design was used. Prior to initiation, electronic invitations detailing the research purpose, ethical principles, and academic use of outcomes were sent to the administrative departments of each participating institution via nursing managers, and institutional approvals were obtained. With the assistance of nursing managers, questionnaires were disseminated to nurses via an online survey platform (https://www.wjx.cn). The online questionnaire was programmed to require a response to every item before submission, thereby preventing incomplete submissions. A pilot survey was carried out before the formal investigation to assess the comprehensibility of questionnaire items and technical feasibility. During the formal survey, uniformly trained investigators explained the study purpose, relevant concepts, and precautions to potential participants, clearly stating that participation was based on voluntary, informed consent and anonymity. Regarding sample size, latent profile analysis requires an adequate sample to ensure the stability and credibility of identified profiles [20]. Simulation studies indicate that reliable model convergence demands a sample size well above the minimum threshold of 300, while accurate identification of cluster numbers necessitates an even larger sample [21, 22]. The target sample size in this study was set with careful consideration and aimed to exceed these methodological recommendations. A total of 1100 questionnaires were distributed. After data collection, responses were excluded for the following reasons: refusal to complete (*n* = 62), selecting the same option for all items across an entire scale (*n* = 18), and logically contradictory responses (e.g., years of nursing experience exceeding age) (*n* = 10). Accordingly, 90 responses were excluded, resulting in 1010 valid questionnaires and an effective response rate of 91.82%.

### 3.2. Participants

This study was conducted in 10 tertiary general hospitals in Sichuan Province, China, from April 9 to June 21, 2025. This decision was made to enhance sample homogeneity by reducing variation introduced by different hospital tiers. A total of 1010 nurses were recruited, all of whom met the following inclusion criteria: (a) registered nurses holding valid practice certificates; (b) full‐time employment with a total clinical work experience of at least one year; (c) being on duty during the survey period; and (d) provision of informed consent and voluntary participation. Exclusion criteria comprised: (a) nurses undergoing internship or standardized training; and (b) those clinically diagnosed with mental disorders or receiving related psychotropic medication within the past 6 months.

### 3.3. Measures

#### 3.3.1. Sociodemographic Characteristics Questionnaire

The sociodemographic characteristics questionnaire included individual characteristics (gender, age, education level, marital status, weekly exercise, and daily sleep hours) and work‐related characteristics (weekly work hours, years of nursing experience, professional title, main shift, and workplace violence). Workplace violence was defined as incidents in which nurses experienced physical assault, verbal abuse, threats, or sexual harassment from patients, family members, or colleagues during work within the past 12 months (dichotomous: yes/no).

#### 3.3.2. The Chinese Version of the Professional Quality of Life Scale (ProQOL‐CN)

The original ProQOL scale was developed by Stamm [13] and was subsequently translated and revised by Zheng et al. [23] to form the ProQOL‐CN. This scale consists of 30 items, which are divided into three subscales: compassion satisfaction, burnout, and secondary traumatic stress, each containing 10 items. A 5‐point Likert scale is used for scoring (1 = “never” to 5 = “always”), with Items 1, 4, 15, 17, and 29 being reverse‐scored. Compassion satisfaction is regarded as the positive subscale, with higher scores indicating greater levels of compassion satisfaction, while burnout and secondary traumatic stress are considered negative subscales, where higher scores reflect greater levels of compassion fatigue. Based on established cut‐off points for each subscale, scores ≤ 22 are classified as low, scores between 23 and 41 as moderate, and scores ≥ 42 as high [15]. In this study, Cronbach’s *α* coefficients for compassion satisfaction, burnout, and secondary traumatic stress subscales were 0.922, 0.823, and 0.755, respectively.

#### 3.3.3. The Simplified Chinese Version of the Posttraumatic Growth Inventory (C‐PTGI)

The original Posttraumatic Growth Inventory (PTGI), developed by Tedeschi and Calhoun [10], was subsequently translated and revised into the C‐PTGI by Wang et al. [24]. The C‐PTGI consists of five dimensions: relating to others, new possibilities, personal strength, spiritual change, and insights on life, encompassing a total of 20 items (the item “I have a stronger religious faith” was excluded). Responses are recorded using a 6‐point Likert scale ranging from 0 (“*not experienced a change*”) to 5 (“*change to a very great extent*”). According to established cutoff criteria, a total score < 60 indicates low posttraumatic growth, a score between 60 and 66 reflects a moderate level, and a score > 66 represents high posttraumatic growth [24]. In this study, Cronbach’s *α* coefficient for the C‐PTGI was 0.972.

### 3.4. Data Analysis

Data analysis was conducted using SPSS 26.0 and Mplus 8.3. Latent profile analysis of nurses’ ProQOL was performed with Mplus 8.3, in which models with one to five classes were sequentially fitted. Model fit was comprehensively evaluated using the following criteria: Akaike information criterion (AIC), Bayesian information criterion (BIC), and adjusted Bayesian information criterion (aBIC), with lower values indicating better fit [18]. Entropy was employed to reflect classification accuracy, where values closer to 1 denote clearer classification, and a threshold of ≥ 0.80 was adopted to indicate good classification [18, 22]. Additionally, the Lo–Mendell–Rubin test (LMR) and the bootstrapped likelihood ratio test (BLRT) were used, with significant *p* values (*p* < 0.05) for both tests, suggesting that the *k* class model was superior to the k‐1 class model [25]. Following the recommendation of Sinha et al. [19], it was ensured that each latent class comprised no less than 10% of the total sample. After identifying the optimal profile model, we used the three‐step approach to examine associations between the latent profiles and covariates, as this approach properly accounts for classification uncertainty and produces less biased estimates than the classify–analyze approach [26]. Specifically, the R3STEP command in Mplus 8.3 was used to estimate the effects of covariates on latent class membership. For the distal outcome (posttraumatic growth), the Bolck–Croon–Hagenaars (BCH) method was applied to compare posttraumatic growth scores across profiles with bias adjustment. For descriptive purposes, SPSS 26.0 was utilized for frequencies, percentages, and preliminary group comparisons (chi‐square tests, *t* tests, and one‐way ANOVA). All analyses were two‐tailed, with the significance level set at *α* = 0.05.

### 3.5. Ethical Considerations

The principles of anonymity and informed consent were strictly followed throughout the study, and this study has been approved by the Ethics Committee of West China Hospital, Sichuan University (No. 20221332). All procedures were performed in accordance with the Declaration of Helsinki.

## 4. Results

### 4.1. Common Method Bias Test

Common method bias was checked with Harman’s single‐factor test. An unrotated exploratory factor analysis yielded a Kaiser–Meyer–Olkin (KMO) value of 0.963 and Bartlett’s test was significant (*χ* ^2^ = 40,032.438, *p* < 0.05). Seven factors showed eigenvalues above 1, and the first factor accounted for 34.05% of the variance, which is below the 40% threshold, suggesting that common method bias was not a major concern.

### 4.2. Participant Characteristics

A total of 1010 nurses participated in this study. The majority were female (91.49%), and more than half of the participants were aged 25–35 years (51.39%). Most nurses held an undergraduate degree or above (76.14%), and 53.47% were married. Additionally, 28.81% engaged in weekly exercise, and 58.42% reported sleeping ≥ 7 h per day. Regarding work‐related characteristics, 66.14% worked > 40 h per week, 48.61% had ≤ 5 years of nursing experience, 44.75% were senior nurses, and 63.56% primarily worked day shifts. A significant proportion (66.24%) reported having experienced workplace violence (Table 1).

**TABLE 1:** Comparison of ProQOL latent profiles by sociodemographic characteristics (*n* = 1010).

Variables	*n* (%)	Depleted	Balanced	Thriving	*χ* ^2^	*p*
		*n* (%)	*n* (%)	*n* (%)		
Gender					5.271	0.072
Male	86 (8.51)	14 (16.28)	60 (69.77)	12 (13.95)		
Female	924 (91.49)	112 (12.12)	586 (63.42)	226 (24.46)		
Age (years)					18.341	**0.001**
< 25	314 (31.09)	43 (13.69)	205 (65.29)	66 (21.02)		
25–35	519 (51.39)	56 (10.79)	351 (67.63)	112 (21.58)		
> 35	177 (17.52)	27 (15.25)	90 (50.85)	60 (33.90)		
Education level					4.658	0.097
Junior college or less	241 (23.86)	39 (16.18)	143 (59.34)	59 (24.48)		
Undergraduate or above	769 (76.14)	87 (11.31)	503 (65.41)	179 (23.28)		
Marital status					8.103	**0.017**
Married	540 (53.47)	59 (10.93)	336 (62.22)	145 (26.85)		
Others	470 (46.53)	67 (14.25)	310 (65.96)	93 (19.79)		
Weekly exercise					20.006	**<** **0.001**
Yes	291 (28.81)	23 (7.90)	175 (60.14)	93 (31.96)		
No	719 (71.19)	103 (14.32)	471 (65.51)	145 (20.17)		
Daily sleep hours					32.575	**<** **0.001**
< 7	420 (41.58)	81 (19.29)	257 (61.19)	82 (19.52)		
≥ 7	590 (58.42)	45 (7.63)	389 (65.93)	156 (26.44)		
Weekly work hours					28.498	**<** **0.001**
≤ 40	342 (33.86)	26 (7.60)	205 (59.94)	111 (32.46)		
> 40	668 (66.14)	100 (14.97)	441 (66.02)	127 (19.01)		
Years of nursing experience					17.917	**0.001**
≤ 5	491 (48.61)	64 (13.03)	330 (67.21)	97 (19.76)		
6–10	234 (23.17)	19 (8.12)	159 (67.95)	56 (23.93)		
> 10	285 (28.22)	43 (15.09)	157 (55.09)	85 (29.82)		
Professional title					18.060	**0.001**
Nurse	359 (35.55)	46 (12.81)	232 (64.63)	81 (22.56)		
Senior nurse	452 (44.75)	59 (13.05)	305 (67.48)	88 (19.47)		
Nurse supervisor or above	199 (19.70)	21 (10.55)	109 (54.78)	69 (34.67)		
Main shift					31.307	**<** **0.001**
Night shift	368 (36.44)	72 (19.57)	230 (62.50)	66 (17.93)		
Day shift	642 (63.56)	54 (8.41)	416 (64.80)	172 (26.79)		
Workplace violence					12.433	**0.002**
Experienced	669 (66.24)	95 (14.20)	436 (65.17)	138 (20.63)		
Unexperienced	341 (33.76)	31 (9.09)	210 (61.58)	100 (29.33)		

*Note:* The bold values represent statistically significant *p* values (*p* < 0.05). ProQOL: professional quality of life.

### 4.3. Latent Profiles of ProQOL

Among 1010 nurses, the scores for compassion satisfaction, burnout, and secondary traumatic stress were 32.13 ± 6.84, 28.03 ± 5.68, and 27.32 ± 4.77, respectively. Using compassion satisfaction, burnout, and secondary traumatic stress as exogenous indicators, fit indices for the one‐ to five‐profile models are displayed in Table 2. The AIC, BIC, and aBIC decreased consistently as the number of profiles increased. The three‐profile model exhibited the highest entropy value (0.826), and both the LMR (*p* < 0.001) and the BLRT (*p* < 0.001) were statistically significant, supporting its superiority over the two‐profile model. The four‐profile solution had a nonsignificant LMR (*p* = 0.121), and the five‐profile solution contained a very small class (2%), raising concerns about stability. Furthermore, the average latent profile assignment probabilities for the three‐profile model ranged from 89.8% to 93.0% (Table 3), confirming high classification accuracy and distinct profile separation. Consequently, the three‐profile solution was retained as optimal.

**TABLE 2:** Fit statistics for each profile structure (*n* = 1010).

Model	AIC	BIC	aBIC	*p* (LMR)	*p* (BLRT)	Entropy	Class proportions
1	19,156.980	19,186.486	19,167.429	—	—	—	—
2	18,714.056	18,763.233	18,731.472	**<** **0.001**	**<** **0.001**	0.656	0.60/0.40
3	18,328.261	18,397.109	18,352.644	**<** **0.001**	**<** **0.001**	0.826	0.12/0.64/0.24
4	18,197.847	18,286.366	18,229.196	0.121	**<** **0.001**	0.789	0.31/0.46/0.12/0.10
5	18,088.587	18,196.776	18,126.902	**0.040**	**<** **0.001**	0.820	0.12/0.10/0.44/0.32/0.02

*Note:* The bold values represent statistically significant *p* values (*p* < 0.05). Class proportions represent the proportion of the sample assigned to each profile.

**TABLE 3:** Average latent class probability matrix for the three‐profile solution (*n* = 1010).

Model	Probability of the latent profile (%)		
	1	2	3
1	0.903	0.097	0.000
2	0.046	0.930	0.024
3	0.000	0.102	0.898

*Note:* ProQOL: professional quality of life. Values on the diagonal represent the probability that nurses assigned to a profile actually belong to that profile; values on the off‐diagonals represent misclassification probabilities.

**FIGURE 1:**
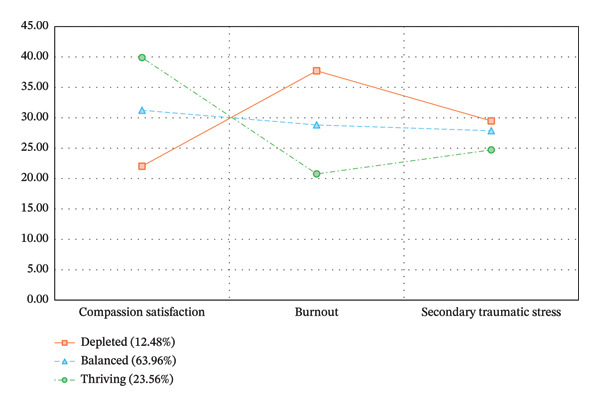
The latent profiles of ProQOL among nurses. ProQOL: professional quality of life.

As illustrated in Figure 1, three latent profiles were identified. While the profiles followed a gradient from resource‐depleted to resource‐abundant, they also showed differences in the relative configuration across the three dimensions. Profile 1 (12.48%, *n* = 126) was characterized by the lowest compassion satisfaction and the highest burnout and secondary traumatic stress scores, and was labelled the “Depleted.” Profile 2 (63.96%, *n* = 646) demonstrated moderate levels across all three subscales and was named the “Balanced.” Profile 3 (23.56%, *n* = 238) exhibited the highest compassion satisfaction and the lowest burnout and secondary traumatic stress scores, and was identified as the “Thriving.”

### 4.4. Characteristics of Latent Profiles of ProQOL and Posttraumatic Growth

Comparisons of sociodemographic characteristics across the three latent profiles revealed statistically significant differences in age, marital status, weekly exercise, daily sleep hours, weekly work hours, years of nursing experience, professional title, main shift, and workplace violence experience. Additionally, nurses who were married, exercised weekly, and slept ≥ 7 h per day had higher posttraumatic growth scores. Details are provided in Tables 1 and 4.

**TABLE 4:** Comparison of posttraumatic growth by sociodemographic characteristics (*n* = 1010).

Variables	*n* (%)	Posttraumatic growth	*t/F*	*p*
		Mean ± SD		
Gender			1.027	0.305
Male	86 (8.51)	54.49 ± 21.18		
Female	924 (91.49)	56.99 ± 21.66		
Age (years)			1.846	0.158
< 25	314 (31.09)	55.97 ± 20.91		
25–35	519 (51.39)	56.31 ± 21.94		
> 35	177 (17.52)	59.59 ± 21.81		
Education level			0.613	0.540
Junior college or less	241 (23.86)	56.03 ± 22.11		
Undergraduate or above	769 (76.14)	57.01 ± 21.47		
Marital status			2.095	**0.036**
Married	540 (53.47)	58.11 ± 21.54		
Others	470 (46.53)	55.25 ± 21.63		
Weekly exercise			3.067	**0.002**
Yes	291 (28.81)	60.04 ± 20.92		
No	719 (71.19)	55.46 ± 21.77		
Daily sleep hours			2.853	**0.004**
< 7	420 (41.58)	54.49 ± 22.26		
≥ 7	590 (58.42)	58.41 ± 21.02		
Weekly work hours			0.328	0.743
≤ 40	342 (33.86)	57.09 ± 22.12		
> 40	668 (66.14)	56.62 ± 21.38		
Years of nursing experience			2.342	0.097
≤ 5	491 (48.61)	55.70 ± 21.13		
6–10	234 (23.17)	56.21 ± 22.72		
> 10	285 (28.22)	59.10 ± 21.42		
Professional title			2.486	0.084
Nurse	359 (35.55)	56.61 ± 20.95		
Senior nurse	452 (44.75)	55.62 ± 22.38		
Nurse supervisor or above	199 (19.70)	59.70 ± 20.90		
Main shift			1.435	0.151
Day shift	642 (63.56)	57.52 ± 21.38		
Night shift	368 (36.44)	55.49 ± 22.00		
Workplace violence			1.706	0.088
Experienced	669 (66.24)	55.95 ± 21.21		
Unexperienced	341 (33.76)	58.40 ± 22.34		

*Note:* The bold values represent statistically significant *p* values (*p* < 0.05).

### 4.5. Factors Associated With Nurses’ ProQOL Latent Profiles

Using the R3STEP method, which accounts for classification uncertainty, we examined factors associated with ProQOL latent profile membership, with the “Thriving” profile as the reference group (Table 5). The results showed that married nurses had lower odds of belonging to the “Depleted” profile (OR = 0.443, 95% CI: 0.208–0.943, *p* = 0.035). Nurses who exercised weekly were less likely to belong to both the “Depleted” (OR = 0.344, 95% CI: 0.172–0.687, *p* = 0.003) and “Balanced” (OR = 0.600, 95% CI: 0.401–0.897, *p* = 0.013) profiles. Sleeping < 7 h per day was associated with higher odds of being in the “Depleted” profile (OR = 2.662, 95% CI: 1.502–4.718, *p* = 0.001). Working ≤ 40 h per week was associated with lower odds of being in both the “Depleted” (OR = 0.353, 95% CI: 0.189–0.658, *p* = 0.001) and “Balanced” (OR = 0.533, 95% CI: 0.364–0.780, *p* = 0.001) profiles. In addition, nurses with 6–10 years of nursing experience had significantly lower odds of being in the “Depleted” profile (OR = 0.382, 95% CI: 0.153–0.954, *p* = 0.039). Nurses who worked night shifts were more likely to be in the “Depleted” profile (OR = 2.401, 95% CI: 1.339–4.307, *p* = 0.003). Experiencing workplace violence was associated with higher odds of being in both the “Depleted” (OR = 2.349, 95% CI: 1.292–4.271, *p* = 0.005) and “Balanced” (OR = 1.761, 95% CI: 1.202–2.581, *p* = 0.004) profiles.

**TABLE 5:** Multinomial logistic regression of ProQOL latent profiles using the R3STEP method (*n* = 1010).

Variables	Depleted				Balanced			
	*β*	SE	*p*	OR (95%CI)	*β*	SE	*p*	OR (95%CI)
Age (years)								
< 25	−0.270	0.633	0.669	0.763 (0.221, 2.640)	0.212	0.477	0.656	1.236 (0.485, 3.148)
25–35	−0.358	0.479	0.455	0.699 (0.273, 1.787)	0.351	0.360	0.329	1.421 (0.701, 2.878)
> 35 (reference)								
Marital status								
Married	−0.813	0.385	**0.035**	0.443 (0.208, 0.943)	−0.309	0.255	0.225	0.734 (0.445, 1.210)
Others (reference)								
Weekly exercise								
Yes	−1.068	0.353	**0.003**	0.344 (0.172, 0.687)	−0.511	0.205	**0.013**	0.600 (0.401, 0.897)
No (reference)								
Daily sleep hours								
< 7	0.979	0.292	**0.001**	2.662 (1.502, 4.718)	0.110	0.208	0.597	1.116 (0.742, 1.679)
≥ 7 (reference)								
Weekly work hours								
≤ 40	−1.042	0.318	**0.001**	0.353 (0.189, 0.658)	−0.629	0.194	**0.001**	0.533 (0.364, 0.780)
> 40 (reference)								
Years of nursing experience								
≤ 5	−0.118	0.504	0.815	0.889 (0.331, 2.387)	0.431	0.397	0.278	1.539 (0.706, 3.352)
6–10	−0.962	0.467	**0.039**	0.382 (0.153, 0.954)	0.040	0.327	0.902	1.041 (0.548, 1.976)
> 10 (reference)								
Professional title								
Nurse	0.369	0.518	0.476	1.447 (0.524, 3.995)	0.064	0.377	0.864	1.066 (0.509, 2.233)
Senior nurse	0.806	0.447	0.071	2.239 (0.932, 5.382)	0.430	0.317	0.176	1.537 (0.825, 2.863)
Nurse supervisor or above (reference)								
Main shift								
Night shift	0.876	0.298	**0.003**	2.401 (1.339, 4.307)	0.030	0.224	0.892	1.030 (0.664, 1.599)
Day shift (reference)								
Workplace violence								
Experienced	0.854	0.305	**0.005**	2.349 (1.292, 4.271)	0.566	0.195	**0.004**	1.761 (1.202, 2.581)
Unexperienced (reference)								

*Note:* The bold values represent statistically significant *p* values (*p* < 0.05). ProQOL: professional quality of life. Both depleted and balanced were based on thriving as the reference group.

Abbreviations: CI, confidence intervals; OR, odds ratio; SE, standard error.

### 4.6. Comparison of Posttraumatic Growth Across ProQOL Latent Profiles

Using the BCH method, which adjusts for classification uncertainty, we compared posttraumatic growth across the three ProQOL latent profiles (Table 6). Significant differences were observed across all profiles for the posttraumatic growth total score and all five dimensions (all *p* < 0.001). Pairwise comparisons revealed a clear gradient: the “Thriving” profile had the highest posttraumatic growth scores, followed by the “Balanced” profile, and the “Depleted” profile had the lowest scores.

**TABLE 6:** Comparison of posttraumatic growth across ProQOL latent profiles using the BCH method (*n* = 1010).

Variable	Depleted (*n* = 126)	Balanced (*n* = 646)	Thriving (*n* = 238)	Adjusted BCH *χ* ^2^	*p*	Pairwise comparison
	Mean (SE)	Mean (SE)	Mean (SE)			
Posttraumatic growth	38.256 (2.031)	54.857 (0.832)	71.516 (1.491)	185.774	< 0.001	Thriving > Balanced > Depleted
Relating to others	11.604 (0.737)	18.430 (0.295)	24.540 (0.532)	212.790	< 0.001	Thriving > Balanced > Depleted
New possibilities	7.781 (0.568)	12.563 (0.215)	16.831 (0.389)	185.364	< 0.001	Thriving > Balanced > Depleted
Personal strength	7.952 (0.428)	10.600 (0.169)	14.166 (0.304)	162.664	< 0.001	Thriving > Balanced > Depleted
Spiritual change	3.552 (0.253)	4.601 (0.088)	5.243 (0.165)	32.248	< 0.001	Thriving > Balanced > Depleted
Insights on life	7.368 (0.382)	8.663 (0.129)	10.735 (0.234)	77.926	< 0.001	Thriving > Balanced > Depleted

*Note:* The bold values represent statistically significant *p* values (*p* < 0.05). ProQOL: professional quality of life. All pairwise differences were statistically significant.

### 4.7. Regression Analysis of ProQOL Latent Profiles on Posttraumatic Growth

Hierarchical linear regression was conducted to examine the relationship between ProQOL latent profiles and posttraumatic growth (Table 7). Model 1 indicated that significant sociodemographic characteristics (marital status, weekly exercise, and daily sleep hours) had limited explanatory power for posttraumatic growth. After adding ProQOL latent profiles in Model 2, the explained variance increased substantially by 14.8%. Specifically, compared with the “Thriving” profile, both the “Depleted” and “Balanced” profiles were significantly negatively correlated with posttraumatic growth.

**TABLE 7:** Hierarchical linear regression analysis of ProQOL latent profiles on posttraumatic growth (*n* = 1010).

	Variables	Β (95% CI)	SE	*β*	*t*	*p*	*R* ^2^	Adjusted *R* ^2^	Δ*R* ^2^	*F*	*p*
Model 1	Marital status						0.021	0.018		7.130	**< 0.001**
	Married	3.213 (0.551, 5.876)	1.357	0.074	2.369	**0.018**					
	Others (reference)										
	Weekly exercise										
	Yes	4.124 (1.180, 7.067)	1.500	0.086	2.749	**0.006**					
	No (reference)										
	Daily sleep hours										
	< 7	−3.735 (−6.450, −1.020)	1.384	−0.085	−2.699	**0.007**					
	≥ 7 (reference)										

Model 2	Marital status						0.169	0.165	0.148	40.824	**< 0.001**
	Married	1.470 (−0.999, 3.938)	1.258	0.034	1.168	0.243					
	Others (reference)										
	Weekly exercise										
	Yes	1.824 (−0.913, 4.561)	1.395	0.038	1.308	0.191					
	No (reference)										
	Daily sleep hours										
	< 7	−1.138 (−3.681, 1.405)	1.296	−0.026	−0.878	0.380					
	≥ 7 (reference)										
	ProQOL										
	Depleted	−29.016 (−33.402, −24.631)	2.235	−0.444	−12.983	**< 0.001**					
	Balanced	−14.257 (−17.226, −11.288)	1.513	−0.317	−9.423	**< 0.001**					
	Thriving (reference)										

*Note:* The bold values represent statistically significant *p* values (*p* < 0.05). CI: confidence intervals. ProQOL: professional quality of life. Δ*R* ^2^ represents the change in *R* ^2^ (Model 2’s *R* ^2^ minus Model 1’s *R* ^2^).

## 5. Discussion

### 5.1. The Level and Latent Profile Characteristics of Nurses’ ProQOL

This study found that nurses’ levels of compassion satisfaction, burnout, and secondary traumatic stress were all at moderate levels, which aligns with the global trend of nurses’ ProQOL as reported by Xie et al. [14]. More importantly, moving beyond the traditional variable‐centered approach, latent profile analysis was employed to identify three profiles of nurses’ ProQOL: “Depleted,” “Balanced,” and “Thriving.” These patterns, derived from self‐reported data at a single time point, should be interpreted as empirical groupings rather than fixed individual traits. Among these, “Balanced” was the largest (63.96%), suggesting a pattern where positive and negative experiences are relatively balanced, which may reflect general adaptation to current work intensity [12]. “Thriving” accounted for 23.56%, characterized by the highest compassion satisfaction and the lowest burnout and secondary traumatic stress, a pattern associated with gaining a sense of achievement and accumulating positive psychological resources [15]. In contrast, “Depleted” (12.48%) was the smallest and exhibited an opposite pattern, highlighting a subgroup with severe professional depletion that may require closer attention from managers [27]. Furthermore, these three patterns lie on a continuum ranging from resource‐abundant to resource‐depleted, consistent with the resource gain and loss dynamics described by conservation of resources theory [16, 27]. These cross‐sectional findings offer preliminary insights for nursing managers to better understand within‐group heterogeneity in nurses’ ProQOL and to consider tailored support strategies.

### 5.2. Factors Associated With Nurses’ ProQOL Latent Profiles

This study found that individual characteristics (marital status, weekly exercise, and daily sleep hours) and work‐related characteristics (weekly work hours, years of nursing experience, main shift, and workplace violence) were significantly associated with nurses’ ProQOL latent profiles.

Adequate personal resources serve as a crucial buffer against professional depletion. This study identified that being married, sleeping ≥ 7 h per day, and weekly exercise were associated with lower odds of falling into “Depleted.” Previous research suggests that married nurses may possess richer social resources and may be more likely to receive support, potentially developing better coping strategies to withstand occupational stress [28, 29]. Sleep, as a fundamental physiological process, restores cognitive and emotional resources, with adequate sleep being associated with maintaining nurses’ mental health [30, 31]. Regular exercise may contribute to emotion regulation and alleviate the physical and psychological stress experienced by nurses in clinical practice [32]. Thus, a stable marital status combined with a healthy lifestyle may jointly provide nurses with resource reserves to cope with work‐related stress.

Furthermore, reasonable work schedules and a supportive work environment may promote resource gain. The results demonstrated that working ≤ 40 h per week and being on day shifts were associated with significantly lower risk of being classified into “Depleted.” Excessive working hours may be directly associated with overconsumption of resources such as energy and time [33], while night shift work may disrupt circadian rhythms and social support systems, potentially exacerbating resource loss [34]. In contrast, a manageable workload and consistent shift patterns may facilitate resource conservation and recovery. Longer years of nursing experience have also been identified as a protective factor, as more experienced nurses tend to develop greater coping skills and psychological resilience, which may buffer against professional depletion [35]. On the other hand, exposure to workplace violence was identified as a risk factor associated with nurses being in the “Depleted” or “Balanced.” Violent incidents themselves may represent a significant resource shock, which may heighten nurses’ vulnerability to stress and may be associated with a resource loss spiral [15, 36]. Collectively, these findings suggest that improving nurses’ ProQOL may benefit from a dual approach focusing on both individual resource cultivation and organizational environment optimization.

### 5.3. The Relationship Between Nurses’ ProQOL Latent Profiles and Posttraumatic Growth

Unlike previous studies focusing on single catastrophic events, the trauma exposure faced by nurses is often characterized by recurrence, continuity, and cumulativeness, while stressors frequently encountered in daily practice, such as patient suffering, end‐of‐life care, workplace violence, and high workloads, collectively constitute a context of “cumulative occupational trauma” unique to nurses [11, 37]. Against this realistic background, this study employs an integrated framework combining posttraumatic growth theory [10] and conservation of resources theory [16] to examine the relationship between ProQOL profiles and posttraumatic growth. The findings indicate that distinct ProQOL latent profiles showed significant associations with nurses’ posttraumatic growth levels, with the explanatory power remaining at 14.8% after controlling for sociodemographic characteristics. Specifically, nurses with the “Thriving” pattern exhibited the highest level of posttraumatic growth. According to posttraumatic growth theory, growth requires that individuals possess sufficient psychological resources, such as the pleasure and sense of accomplishment derived from compassion satisfaction, to engage in profound cognitive processing and meaning reconstruction after experiencing highly challenging events [8, 15]. Conservation of resources theory further suggests that nurses characterized by “Thriving” may be in a state of resource gain. Their abundant personal resources, such as positive emotions, may not only buffer the damage caused by stress but also be associated with resource gains that could, in turn, correlate with growth, potentially enhancing their capacity to identify new possibilities and gain personal strength from challenges [16, 38]. Conversely, nurses with a “Depleted” show the lowest level of posttraumatic growth, which may indicate that they could be trapped in a resource loss spiral. That is, high burnout and secondary traumatic stress may continually deplete nurses’ emotional and cognitive resources, potentially making it difficult to initiate or sustain the cognitive and meaning‐making processes necessary for posttraumatic growth [17, 39]. Additionally, nurses with “Balanced” represent an intermediate state, where moderate resource levels may allow for some degree of personal growth, yet this remains substantially lower than that of “Thriving” [12]. Therefore, this gradient relationship between profiles and growth highlights the potential limitation of viewing nurses as a homogeneous group and suggests the potential value of implementing differentiated psychological support strategies based on distinct profile characteristics.

It is worth noting that although the BCH method was used to correct for classification uncertainty, making cross‐profile comparisons more reliable, for certain subdimensions (e.g., spiritual change), the mean differences across profiles, while statistically significant, are relatively small in magnitude. This finding suggests that we need to distinguish between statistical significance and clinical significance when interpreting the results. In nursing practice, even modest differences in spiritual change, if accumulated at the group level, may still have important implications for nurses’ overall well‐being and sense of professional meaning. Therefore, when developing intervention strategies, managers should consider not only the magnitude of effect sizes but also the cumulative value and long‐term significance of these differences in real‐world clinical settings.

Interestingly, in the hierarchical regression, marital status, weekly exercise, and daily sleep hours were significantly associated with posttraumatic growth in Model 1 but became non‐significant after adding ProQOL profiles in Model 2. This pattern suggests that ProQOL profiles may either (a) mediate the relationships between these factors and posttraumatic growth, or (b) capture variance that substantially overlaps with these sociodemographic variables. In other words, the beneficial associations of these factors with posttraumatic growth may operate, at least partly, through their link to more favorable ProQOL profiles. Because of the cross‐sectional design, we cannot formally test mediation. However, this finding highlights the central role of ProQOL patterns as an integrative construct in understanding nurses’ psychological growth.

### 5.4. Limitations

This study has several limitations. Firstly, the cross‐sectional design precludes causal inference regarding the relationship between ProQOL profiles and posttraumatic growth. The reported associations should be interpreted as correlational, not directional. Future research should employ longitudinal designs to track changes in profile stability and their temporal effects on growth trajectories. Secondly, the sample was drawn from 10 tertiary hospitals in Sichuan Province. While of considerable size, it may be influenced by regional culture, hospital policies, and the fact that all hospitals were tertiary‐level, limiting the generalizability of the findings to nurses in lower‐tier hospitals or other regions. Additionally, potential clustering effects at the hospital level were not modeled, as our primary research question focused on individual‐level ProQOL patterns. Future multicenter and cross‐cultural studies, including hospitals of different tiers, are needed to verify the universality of these profiles. Thirdly, all data were collected using self‐report measures, which may introduce common method bias and social desirability bias. Although Harman’s single‐factor test indicated that common method bias was not severe, this test has known limitations (low sensitivity). Future research could incorporate objective indicators (e.g., sick leave records, turnover rates) or other‐rated data (e.g., supervisor evaluations) for multimethod validation. Fourth, posttraumatic growth was administered without identifying a specific index traumatic event. Thus, our findings may reflect growth associated with cumulative stress rather than a single trauma, which should be considered when interpreting results. Fifth, the lack of collected data on the frequency, severity, or types of workplace violence may have differential effects on ProQOL and posttraumatic growth, and their absence limits the nuanced interpretation of the role of workplace violence. Finally, residual confounding may be present, as not all potential confounders (e.g., personality traits and social support) were measured.

## 6. Conclusions

This study applied latent profile analysis to identify three distinct profiles of nurses’ ProQOL: “Depleted,” “Balanced,” and “Thriving.” These profiles were associated with individual characteristics, such as marital status, weekly exercise, and daily sleep hours, along with work‐related characteristics, including weekly work hours, main shift, and workplace violence. Furthermore, a graded association was observed between these profiles and nurses’ levels of posttraumatic growth. Specifically, the “Thriving” pattern, representing resource abundance, was associated with the highest posttraumatic growth level, whereas the “Depleted” pattern, representing resource depletion, was linked to the lowest. These cross‐sectional findings suggest that improving nurses’ psychological well‐being may require differentiated strategies rather than a uniform approach. Given the cross‐sectional design, causal inferences cannot be drawn, and these suggested directions require validation in longitudinal or interventional studies.

## 7. Implications for Nursing Management

These findings provide a preliminary perspective for administrators to recognize within‐group heterogeneity in nurses’ ProQOL. Based on these cross‐sectional associations, the following strategies may be considered for future development and testing: building a resource‐supportive environment through measures such as monitoring work hours to prevent excessive overtime, optimizing shift schedules, and establishing zero‐tolerance policies for workplace violence. Additionally, differentiated psychological support strategies could be explored: for example, providing resource‐replenishing support (e.g., counseling and workload reduction) for nurses in the “Depleted” pattern, and offering growth‐enabling guidance (e.g., peer support and meaning‐focused workshops) for those in the “Balanced” pattern. It is important to note that these suggestions are derived from cross‐sectional data and should be interpreted as hypotheses for future intervention research rather than proven recommendations. Consequently, future longitudinal or quasiexperimental studies are needed to test whether nurses can be better supported to transform occupational challenges into personal growth, which could ultimately promote workforce stability and enhance the quality of nursing care.

## Author Contributions

Li Zeng: writing–review and editing, writing–original draft, methodology, data curation, and conceptualization. Yuan Zhang: investigation and data curation. Zhongqing Yuan: formal analysis and data curation. Fengxue Yang: validation and methodology. Xiuying Hu: conceptualization, supervision, and methodology.

## Funding

This study was supported by the Research Planning Project of the Sichuan Psychological Society (Number: SCSXLXH202403012).

## Ethics Statement

The principles of anonymity and informed consent were strictly followed throughout the study, and this study has been approved by the Ethics Committee of West China Hospital, Sichuan University (No. 20221332).

## Conflicts of Interest

The authors declare no conflicts of interest.

## Data Availability

All raw data generated or analyzed during this study are available from the corresponding author upon reasonable request.
